# Relationship Between Preoperative Arthrogenic Muscle Inhibition Grade and Knee Functional Outcome Following Anterior Cruciate Ligament Reconstruction

**DOI:** 10.7759/cureus.108698

**Published:** 2026-05-12

**Authors:** Sarah Baig, Rohit K R, Sai Aditya Raman, Sai Vineet Damodar Premkumar, Thiagarajan K A, Arumugam Sivaraman, Nithila Sundresh, Hemand S R

**Affiliations:** 1 Department of Arthroscopy and Sports Medicine, Sri Ramachandra Institute of Higher Education and Research, Chennai, IND

**Keywords:** anterior cruciate ligament reconstruction, arthrogenic muscle inhibition, ikdc subjective knee form, meniscal injury, prehabilitation, quadriceps inhibition, santi classification

## Abstract

Introduction

Arthrogenic muscle inhibition (AMI) is a reflexive reduction in quadriceps activation following knee injury that may persist into the perioperative period. Decreased preoperative quadriceps function is thought to contribute to poor postoperative knee functional outcomes. This study aimed to determine the relationship between preoperative AMI grade and International Knee Documentation Committee (IKDC) score three months after anterior cruciate ligament (ACL) reconstruction.

Materials and methods

This single-center retrospective cohort study included 82 adults undergoing ACL reconstruction stratified into two groups - one comprising patients with isolated ACL injury (n = 28) and the other group comprising patients with concomitant meniscal injury (n = 54). Preoperative AMI was graded using the Scientific Anterior Cruciate Ligament Network International (SANTI) clinical classification, and the primary outcome was the IKDC Subjective Knee Form score at three months postoperative. Preoperative AMI grade was modeled as an ordinal variable in multivariable linear regression to assess its association with three-month IKDC, adjusting for preoperative IKDC and time since injury. The same regression model was first applied to the whole cohort and then repeated separately within the isolated ACL and ACL with meniscal injury subgroups. Statistical analyses were performed using IBM SPSS Statistics for Windows, version 29.0 (IBM Corp., Armonk, NY, USA).

Results

Preoperative AMI grade for all 82 patients showed weak negative correlation with three-month IKDC score (r = −0.27, p = 0.014). In adjusted regression, higher AMI grade was associated with lower three-month IKDC (β = −1.51; 95% confidence interval (CI), −2.46 to −0.56; p = 0.002). In the ACL plus meniscal injury subgroup, the association was more pronounced (β = −2.43; 95% CI, −3.61 to −1.25; p < 0.001). No significant association was observed in isolated ACL (β = +0.80; 95% CI, −1.07 to +2.66; p = 0.386).

Conclusions

Higher preoperative AMI grade was associated with lower three-month IKDC after ACL reconstruction. The association was stronger in patients with concomitant meniscal pathology. Preoperative AMI grading may identify patients at risk of poorer early functional recovery and support the rationale for targeted preoperative neuromuscular rehabilitation.

## Introduction

Anterior cruciate ligament (ACL) reconstruction is among the most common orthopedic sports procedures, yet early postoperative recovery remains variable. One clinically important contributor is arthrogenic muscle inhibition (AMI), a reflexive reduction in voluntary quadriceps activation that is driven by altered afferent input from the injured joint and may be influenced by effusion, pain, inflammation, and supraspinal changes [1-4]. Clinically, AMI presents as poor vastus medialis obliquus activation, extensor lag, or inability to maintain terminal knee extension.

The Scientific Anterior Cruciate Ligament Network International (SANTI) clinical classification translates this phenomenon into a practical bedside grading system applicable during routine evaluation, with excellent inter-rater and intra-rater reliability reported in the ACL setting [5,6]. Preoperative quadriceps strength has been shown to predict knee function after ACL reconstruction [7,8], and AMI represents a clinically accessible proxy for that neuromuscular status. If preoperative AMI grade is associated with early postoperative function, it may help guide patient counseling and identify candidates for targeted preoperative neuromuscular rehabilitation.

Concomitant meniscal pathology is present in a large proportion of ACL tears [9,10] and adds pain, effusion, and intra-articular afferent stimulation that may sustain or worsen AMI [11,12]. This raises the question of whether the prognostic value of preoperative AMI grade is greater in patients with combined ACL and meniscal injury than in those with isolated ACL injury.

This study aimed to determine the relationship between preoperative AMI grade and International Knee Documentation Committee (IKDC) score three months after ACL reconstruction, and to investigate whether this relationship differs between isolated ACL injury and ACL with concomitant meniscal injuries. We hypothesized that the association would be stronger in patients with concomitant meniscal pathology, given that meniscal injury adds sustained intra-articular afferent stimulation that maintains quadriceps inhibition beyond that produced by ACL injury alone. Three-month IKDC was selected as the primary endpoint as it represents a clinically relevant early recovery milestone that determines rehabilitation trajectory and is the phase in which preoperative neuromuscular status is most likely to exert measurable influence before rehabilitation effects equalize outcomes over longer follow-up.

## Materials and methods

Study design and ethics

This was a single-center retrospective cohort study of adults undergoing ACL reconstruction with clinical variables recorded during routine care between April and December 2025. The study was conducted in accordance with the Declaration of Helsinki. Ethics approval was obtained from the Institutional Research Ethics Committee, Sri Ramachandra Institute of Higher Education and Research (Protocol Number: CSP-MED/25/FEB/113/41). Written informed consent was obtained from all participants prior to enrollment.

Participants

Of 102 consecutive patients assessed for eligibility, 82 were included in the analytic cohort. Twenty patients were excluded: isolated posterior cruciate ligament injury (n = 7), isolated meniscal injury without ACL tear (n = 6), multiligament injury (n = 3), chondral damage (n = 1), ACL avulsion fracture (n = 1), medial patellofemoral ligament tear (n = 1), and intra-articular loose body (n = 1). The analytic cohort comprised 28 patients with isolated ACL reconstruction and 54 with ACL reconstruction plus concomitant meniscal pathology. The inclusion and exclusion criteria are summarized in Table 1.

**Table 1:** Inclusion and exclusion criteria. ACL: anterior cruciate ligament; AMI: arthrogenic muscle inhibition; IKDC: International Knee Documentation Committee; MRI: magnetic resonance imaging

Inclusion criteria	Exclusion criteria
Age 18 years or older	Multiligament injury
MRI-confirmed full-thickness ACL tear	Intra-articular fracture or avulsion fracture
ACL reconstruction with or without concomitant meniscal pathology confirmed intraoperatively	Isolated non-ACL pathology
Complete records for preoperative AMI grade	Chondral injury as a primary diagnosis
Complete records for preoperative IKDC score	Conditions independently affecting lower-extremity function
Complete records for time since injury	
Complete records for three-month postoperative IKDC score	

Preoperative AMI assessment

Preoperative AMI was graded using the SANTI clinical classification [5,6]. With the patient supine, the examiner assessed voluntary quadriceps contraction, vastus medialis obliquus activation, active knee extension, and response to simple facilitation maneuvers. Grade 0 represents normal activation; grade 1A, inhibited contraction reversible with simple facilitation; grade 1B, inhibited contraction not reversible with simple maneuvers; and grade 2A, inhibited contraction with a reducible extension deficit due to hamstring contracture. No patient in this cohort had grade 2B or grade 3 AMI.

Outcome measure

The primary outcome was the IKDC Subjective Knee Form score (%) at three months postoperatively, collected as part of routine clinical follow-up. The IKDC is a validated patient-reported measure of knee symptoms, function, and activity [13]. The IKDC form is publicly available through the American Orthopaedic Society for Sports Medicine and is available without a license or fee for investigators conducting academic research. The questionnaire was used without modification. Preoperative IKDC was recorded at the initial assessment.

Statistical analysis

Continuous variables are reported as mean (standard deviation, SD) or median (interquartile range, IQR). Between-group comparisons used Welch t-tests for continuous variables, Mann-Whitney U test for time since injury, and Fisher exact test for sex. AMI grade distribution between subgroups was compared using the chi-squared test. AMI grade was modeled ordinally (grade 0 = 0, 1A = 1, 1B = 2, and 2A = 3) as the primary exposure. The primary model used linear regression to estimate the association between preoperative AMI grade and three-month IKDC in the whole cohort, adjusting for preoperative IKDC and time since injury. The same model was then fitted separately within each diagnosis subgroup. An AMI-by-diagnosis interaction term was tested in a combined model. Data were collected and summarized in Microsoft Excel (Microsoft Corp., Redmond, WA, USA). Statistical analyses were performed using IBM SPSS Statistics for Windows, version 29.0 (IBM Corp., Armonk, NY, USA). A p-value of <0.05 was considered statistically significant.

## Results

Cohort characteristics

The cohort was predominantly male (80 of 82, 97.6%) with a mean age of 24.9 years (SD 5.5). Median time since injury was 2.0 months (IQR 1.0-3.0). Mean preoperative IKDC was 63.9% (SD 9.0). Baseline characteristics were similar between subgroups (Table 2). Preoperative IKDC was numerically higher in the ACL plus meniscus group (65.3 vs. 61.1, p = 0.059), reflecting a modest baseline difference that was accounted for by covariate adjustment in all regression models. AMI grade distribution did not differ significantly between subgroups (χ² = 3.75, df = 3, p = 0.290).

**Table 2:** Baseline characteristics by diagnosis subgroup. Values are presented as mean (SD), median (IQR), or n (%) as appropriate. AMI grade distribution was compared between the isolated ACL and ACL plus meniscal injury subgroups using the chi-squared test. AMI: arthrogenic muscle inhibition; ACL: anterior cruciate ligament; IKDC: International Knee Documentation Committee; IQR: interquartile range; SD: standard deviation

Characteristic	Whole cohort (n = 82)	Isolated ACL (n = 28)	ACL + meniscal injury (n = 54)
Age, years, mean (SD)	24.9 (5.5)	24.1 (3.9)	25.3 (6.1)
Male sex, n (%)	80 (97.6%)	28 (100%)	52 (96.3%)
Time since injury, months, median (IQR)	2.0 (1.0-3.0)	2.0 (1.4-3.0)	2.0 (1.0-3.0)
Preoperative IKDC, %, mean (SD)	63.9 (9.0)	61.1 (9.7)	65.3 (8.4)
Three-month IKDC, %, mean (SD)	59.4 (3.1)	59.5 (2.9)	59.4 (3.2)
AMI grade 0, n (%)	8 (9.8%)	3 (10.7%)	5 (9.3%)
AMI grade 1A, n (%)	46 (56.1%)	12 (42.9%)	34 (63.0%)
AMI grade 1B, n (%)	23 (28.0%)	10 (35.7%)	13 (24.1%)
AMI grade 2A, n (%)	5 (6.1%)	3 (10.7%)	2 (3.7%)
AMI distribution, p	χ² = 3.75, df = 3, p = 0.290 (chi-squared test)		

Whole-cohort AMI grade and three-month IKDC

**Figure 1:**
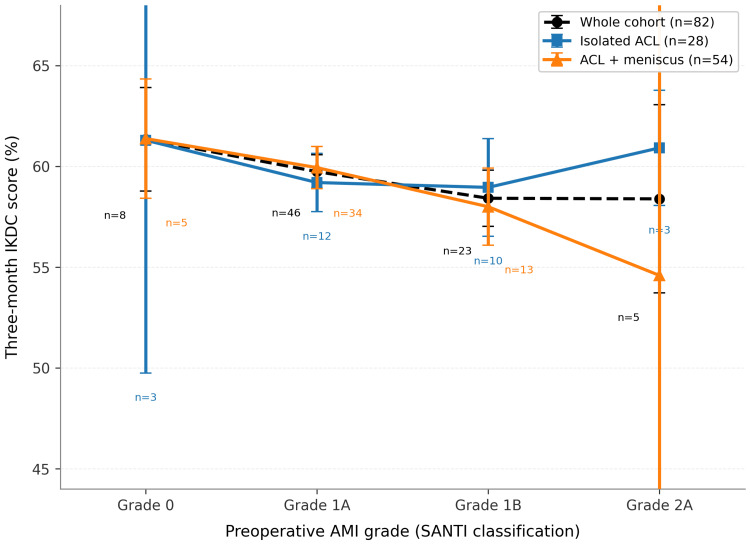
Mean three-month IKDC score (±95% CI) across preoperative AMI grades by diagnosis. Mean three-month IKDC score with 95% confidence intervals (CIs) across preoperative AMI grades for the whole cohort (black dashed line), isolated ACL subgroup (blue), and ACL plus meniscal injury subgroup (orange). Error bars represent 95% CI calculated using the t-distribution. Sample counts per grade are shown for each subgroup. ACL: anterior cruciate ligament; AMI: arthrogenic muscle inhibition; IKDC: International Knee Documentation Committee; SANTI: Scientific Anterior Cruciate Ligament Network International

Across the whole cohort, the mean three-month IKDC score declined with increasing preoperative AMI grade: 61.4 (SD 3.1) at grade 0, 59.7 (SD 2.8) at grade 1A, 58.4 (SD 3.2) at grade 1B, and 58.4 (SD 3.8) at grade 2A (Figure 1). Preoperative AMI grade correlated inversely with three-month IKDC (r = −0.27, p = 0.014; Spearman ρ = −0.23, p = 0.042). In adjusted regression, higher AMI grade remained significantly associated with lower three-month IKDC (β = −1.51 per grade; 95% confidence interval (CI), −2.46 to −0.56; p = 0.002; R² = 0.14), corresponding to a difference of approximately 4.5 IKDC points between grade 0 and grade 2A.

Diagnosis-stratified analysis

In ACL reconstruction with concomitant meniscal pathology (n = 54), a higher preoperative AMI grade was strongly associated with lower three-month IKDC (β = −2.43 per grade; 95% CI, −3.61 to −1.25; p < 0.001; R² = 0.31). Mean three-month IKDC declined monotonically across grades in this subgroup: 61.4 at grade 0, 59.9 at grade 1A, 58.0 at grade 1B, and 54.6 at grade 2A - a difference of approximately 6.8 points across the grade range. Mean IKDC also declined from 65.3 (SD 8.4) preoperatively to 59.4 (SD 3.2) at three months (mean change −5.9%; paired t = 4.77, p < 0.001).

In isolated ACL reconstruction (n = 28), no significant association was observed between preoperative AMI grade and three-month IKDC (β = +0.80 per grade; 95% CI, −1.07 to +2.66; p = 0.386; R² = 0.09). The mean three-month IKDC did not decline consistently across AMI grades in this subgroup, and mean IKDC changed minimally from 61.1 (SD 9.7) preoperatively to 59.5 (SD 2.9) at three months (mean change −1.6%; p = 0.393).

**Figure 2:**
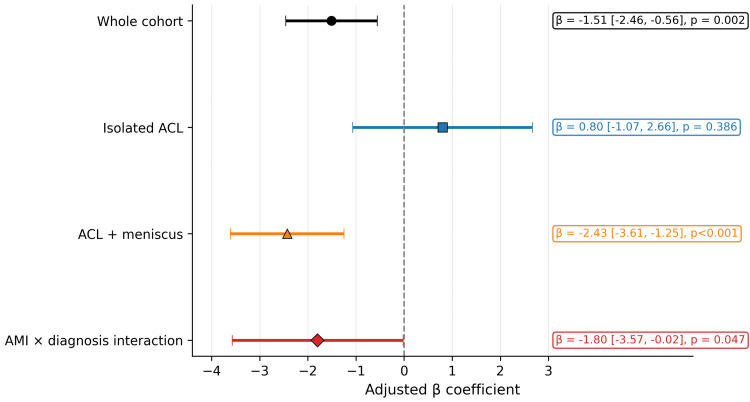
Adjusted β-coefficients and 95% confidence intervals for the association between preoperative AMI grade and three-month IKDC score in the whole cohort, diagnosis subgroups, and AMI-by-diagnosis interaction model. Forest plot showing adjusted β-coefficients and 95% confidence intervals for the association between preoperative AMI grade and three-month IKDC score in the whole cohort and diagnosis subgroups, along with the AMI-by-diagnosis interaction term. Each β reflects the change in three-month IKDC per one-step AMI grade increment, adjusted for preoperative IKDC and time since injury. The dashed vertical line indicates no effect (β = 0). ACL: anterior cruciate ligament; AMI: arthrogenic muscle inhibition; β: regression coefficient; IKDC: International Knee Documentation Committee

In the combined model, the AMI-by-diagnosis interaction term was statistically significant (β = −1.80; 95% CI, −3.57 to −0.02; p = 0.047), confirming that the AMI-IKDC relationship differed by diagnosis subgroup (Figure 2). Regression results across all models are shown in Table 3.

**Table 3:** Adjusted regression models for preoperative AMI grade and three-month IKDC. Linear regression models adjusted for preoperative IKDC and time since injury. AMI was modeled ordinally (grade 0 = 0, 1A = 1, 1B = 2, and 2A = 3). The interaction model additionally included the diagnosis subgroup and the AMI-by-diagnosis interaction term. β reflects the change in three-month IKDC per one-step AMI grade increment. ACL: anterior cruciate ligament; AMI: arthrogenic muscle inhibition; CI: confidence interval; IKDC: International Knee Documentation Committee

Model	n	β (95% CI)	p-value	R²
Whole cohort	82	−1.51 (−2.46 to −0.56)	0.002	0.14
Isolated ACL	28	+0.80 (−1.07 to +2.66)	0.386	0.09
ACL + meniscal injury	54	−2.43 (−3.61 to −1.25)	<0.001	0.31
AMI × diagnosis interaction	82	−1.80 (−3.57 to −0.02)	0.047	0.19

## Discussion

Early functional recovery after ACL reconstruction is influenced by several preoperative and injury-related factors, including quadriceps activation status. In this cohort, higher preoperative AMI grade was associated with lower three-month postoperative IKDC scores, particularly among patients with concomitant meniscal injury. Across the whole cohort, each AMI grade increment corresponded to approximately 1.5 fewer IKDC points at three months after adjustment, a direction consistent with the biological premise that impaired preoperative quadriceps activation carries forward into early postoperative function. The association was most pronounced in the ACL plus meniscal injury subgroup, where the per-grade effect was larger in magnitude and statistically significant (p < 0.001), while no significant association was found in isolated ACL reconstruction. A significant interaction term (p = 0.047) supported a statistically significant difference in the AMI-IKDC association between subgroups.

This aligns with prior evidence that preoperative neuromuscular status matters for postoperative recovery. Eitzen et al. showed that preoperative quadriceps strength predicted IKDC at two years after ACL reconstruction, while Logerstedt et al. reported that preoperative quadriceps strength predicted IKDC2000 scores at six months after reconstruction [7,8]. The present study extends that concept to a simple bedside AMI grade at the initial consultation. Hart et al. described quadriceps activation failure as a common consequence of knee injury, while Lynch et al. demonstrated that activation failure may persist after ACL rupture [3,4]. Together, these findings support the biological plausibility of AMI as a clinically relevant preoperative marker. The SANTI grade captures the clinical expression of this inhibition at a single preoperative examination [5,6].

The stronger association in the meniscal subgroup is biologically coherent. Keyhani et al. and Hagino et al. reported that meniscal tears are commonly associated with ACL injury, supporting the clinical relevance of analyzing this subgroup separately [9,10]. Concomitant meniscal pathology adds sustained intra-articular irritation beyond the ACL injury itself, increasing intra-articular afferent input through pain, effusion, and mechanical disturbance, thereby sustaining reflexive quadriceps inhibition. Torry et al. showed that experimental knee joint effusion reduces quadriceps activation and alters limb mechanics, while Shakespeare et al. demonstrated that quadriceps reflex inhibition after meniscectomy can occur even without an association with perceived pain [11,12]. In this context, a higher preoperative AMI grade in the meniscal subgroup reflects a more sustained inhibitory environment, which translates into greater variability in early outcome and therefore greater prognostic signal.

The absence of significance in isolated ACL should not be read as evidence that AMI is irrelevant in that group. The subgroup was small (n = 28), the three-month IKDC was narrowly distributed (SD 2.9), and only three patients reached grade 2A, leaving the high-grade estimates unstable. There is also a plausible biological reason for a potentially weaker relationship: isolated ACL reconstruction generates less sustained postoperative intra-articular irritation than combined procedures, so AMI may resolve more rapidly and exert less measurable influence on outcomes at three months. Whether this pattern persists in larger isolated ACL cohorts requires further study.

From a practical standpoint, AMI grading requires no specialized equipment and can be incorporated into routine preoperative clinical assessment. For patients with higher grades, particularly those with concomitant meniscal pathology, the findings support the rationale for closer perioperative neuromuscular assessment. Sonnery-Cottet et al. reported moderate-quality evidence for exercise and cryotherapy in reducing quadriceps activation failure after ACL injury or reconstruction, and Hopkins et al. demonstrated experimentally that cryotherapy combined with transcutaneous electrical stimulation can reduce AMI in a joint effusion model [14,15]. Whether improving preoperative AMI grade translates directly into better postoperative IKDC remains to be tested prospectively, but the current data provide a practical grading tool to identify patients who may require closer neuromuscular attention.

This study was limited by its single-center retrospective design and modest sample size, particularly within subgroup analyses. The cohort was predominantly male, reflecting the sport profile and referral patterns of our clinical population, which limits generalizability to female athletes. Although standardized institutional rehabilitation protocols were in place for all patients, individual rehabilitation adherence was not recorded as a variable. Objective quadriceps testing was also not available. The observed associations should be interpreted as correlational rather than causal, and prospective studies with larger and more diverse cohorts are needed to confirm these findings.

## Conclusions

Higher preoperative AMI grade was associated with lower three-month IKDC scores after ACL reconstruction. This association was present in the whole cohort and was more pronounced in patients with concomitant meniscal pathology, while no significant association was observed in isolated ACL reconstruction. Preoperative AMI grading is a simple bedside assessment that may improve early risk stratification and support the rationale for targeted preoperative neuromuscular rehabilitation, particularly in patients with meniscal involvement.
